# A hybrid features fusion-based framework for classification of breast micronodules using ultrasonography

**DOI:** 10.1186/s12880-024-01425-y

**Published:** 2024-09-20

**Authors:** Mousa Alhajlah

**Affiliations:** College of Applied Computer Science, King Saud University, Riyadh, 11543 Saudi Arabia

**Keywords:** Breast cancer detection, Hybrid CNN framework, InceptionV3, MobileNetV2, Computer-aided diagnosis (CAD), Ultrasonography

## Abstract

**Background:**

Breast cancer is one of the leading diseases worldwide. According to estimates by the National Breast Cancer Foundation, over 42,000 women are expected to die from this disease in 2024.

**Objective:**

The prognosis of breast cancer depends on the early detection of breast micronodules and the ability to distinguish benign from malignant lesions. Ultrasonography is a crucial radiological imaging technique for diagnosing the illness because it allows for biopsy and lesion characterization. The user’s level of experience and knowledge is vital since ultrasonographic diagnosis relies on the practitioner’s expertise. Furthermore, computer-aided technologies significantly contribute by potentially reducing the workload of radiologists and enhancing their expertise, especially when combined with a large patient volume in a hospital setting.

**Method:**

This work describes the development of a hybrid CNN system for diagnosing benign and malignant breast cancer lesions. The models InceptionV3 and MobileNetV2 serve as the foundation for the hybrid framework. Features from these models are extracted and concatenated individually, resulting in a larger feature set. Finally, various classifiers are applied for the classification task.

**Results:**

The model achieved the best results using the softmax classifier, with an accuracy of over 95%.

**Conclusion:**

Computer-aided diagnosis greatly assists radiologists and reduces their workload. Therefore, this research can serve as a foundation for other researchers to build clinical solutions.

## Introduction

Currently, the most malignant tumor in the world is breast cancer, which is a highly heterogeneous tumor [1, 2]. The biomarker expression assessed by immunohistochemistry (IHC) was used by the 2013 St. Gallen to divide breast cancer into five subtypes [3]. Breast cancer biomarker expression and various subtypes are significant prognostic factors [4]. After lung, stomach, liver, and colon cancers, breast cancer is the fifth most prevalent cause of cancer-related fatalities in the modern era. It is the leading cause of cancer death in women [5]. In 2005 alone, 519,000 deaths from breast cancer were reported [6]. This indicates that breast cancer caused one in every 100 fatalities globally and nearly one in every fifteen deaths from cancer. Among females, one in four cancer diagnoses is breast cancer, and one in six cancer fatalities is related to breast cancer [7]. Breast cancer also carries a hefty financial cost. The yearly cost of healthcare for women with breast cancer is $13,000 more than that of those without the disease [8]. In 2020, there were 10.9 million cancer-related deaths (9.9 million excluding NMSC, excluding basal cell carcinoma) and 19.3 million new cases of cancer (18.1 million excluding NMSC, excluding basal cell carcinoma) worldwide [7].

A tumor can be classified as malignant (cancerous) or benign (uncancerous). Benign tumors are not harmful because they do not spread cancer; their cells develop slowly, resemble normal tissue, and do not invade nearby tissues or injure other body components. On the other hand, cancerous tumors pose a threat. They eventually grow larger than the initial tumor and target other body areas if they are not treated [9].

One crucial non-radiation imaging method for identifying and categorizing breast tumors is breast ultrasonography. Patients tolerate it well and integrating it into interventional procedures for patient treatments is a simple process [10]. Breast US accuracy depends on the operator’s technical proficiency and experience; however, it is still limited. A standardized vocabulary and reporting system for evaluating breast mass and characterizing its characteristics are provided by the Breast Imaging Reporting and Data System (BI-RADS). Although several BI-RADS US descriptors are linked to both benign and malignant lesions, especially category 4 breast masses, it has been demonstrated to be an excellent approach for discriminating between benign and malignant masses [11].

The identification of breast tumors using combined B-mode and ultrasound elastography is currently limited. The experience of the doctors has a major impact on how ultrasounds operate [11]. It is indisputable that measurement errors result from differences in probe placement/orientation and annotation across and among observers [12, 13]. Furthermore, it can be challenging to determine the boundary between benign and malignant lesions as well as between normal and tumor tissue. Necrosis and liquefaction in malignant lesions or mechanization or calcification in benign lesions might compromise the accuracy of the malignancy rating method [14, 15].

Many computer-aided diagnosis (CAD) systems have been created in the literature for different medical diagnoses [16–18] and to help diagnose breast cancer patients by differentiating between benign and malignant tumors on images [19–21]. These methods have previously been shown to improve diagnostic precision and reduce observer variability [22–24]. Feature extraction, selection, and classification are involved in the classification process when utilizing conventional CAD systems [25, 26]. Effective feature extraction is the main challenge with these systems, as noted by [27], and it has an impact on total performance. Radiologists manually segmented the region of interest (ROI) without using any pre-processing techniques, as reported by Moon et al. [28]. Following various pre-processing methods, manual segmentation was performed in the work presented in [29]. The GAD and response diffusion (RD) based level set segmentation were combined by Zhang et al. [30]. Yu et al.‘s [31] important addition was the pre-processing procedures they took, which included dyadic wavelet transform, active contour, and k-means clustering. A suggested method [32] used a deep learning architecture and included feature extraction, segmentation, and classification, with a focus on Convolutional Neural Networks (CNNs). A variety of CNN models, such as Xception [19], InceptionV3 [21], InceptionNesNetV2 [33], DenseNet1 [20], DenseNet161 [34], and NASNetMobile [20], were tested and compared by Fujioka et al. [35]. Misra et al. [36], in contrast, used two CNN models (AlexNet [37] and ResNet [38]) and used ensemble learning to combine the models with ultrasound modalities (B-mode and SWE). However, even though CNN was used, Zhang et al. [39] and Zhou et al. [40] configured the feature extraction and classifier independently. Many of the recent studies [41–43] have also performed the diagnosis of diseases using various deep-learning techniques and achieved good results in this area. Identifying significant elements in an image requires a lot of work and effort. Furthermore, it might be difficult to optimize conventional CAD’s overall performance.

In this research, we have proposed a Hybrid Convolutional Neural Network (HCNN) system for the diagnosis of benign and malignant breast cancer lesions. The models InceptionV3 and MobilenetV2 serve as the foundation for the hybrid framework. These models’ characteristics are retrieved and concatenated individually. As a result, more features can be utilized. Finally, various classifiers are applied to the classification task.

The remaining structure of the manuscript is as follows: the second section contains details about the dataset as well as the detailed research techniques. The third section highlights the important results of the manuscript along with a discussion of the given results. Finally, the fourth section concludes the research.

## Materials and methods

### Dataset

**Fig. 1:**
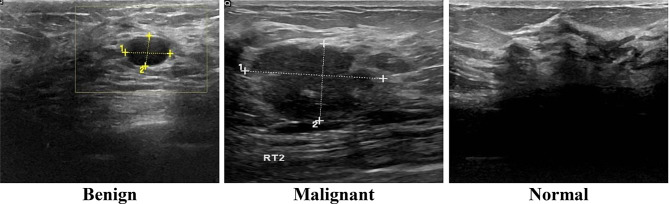
Sample images from dataset

One of the most crucial techniques for determining the sensitivity and efficacy of a screening modality is ultrasonography. The dataset used in this work is publicly available and was published in 2020 [44]. Breast ultrasound images were collected from women between the ages of 25 and 75 as part of the baseline data collection which was collected in 2018. There are 600 female patients in total. The collection contains 780 images, with an average pixel size of 500 by 500. The images are in PNG format. Original images are displayed with the ground truth images. The images are divided into three categories: normal, benign, and malignant. The benign class contains 437 images, malignant contains 210 images, and the normal contains 133 images. Fig. 1 shows the sample images from the ultrasonography images dataset.

### Methodology

**Fig. 2:**
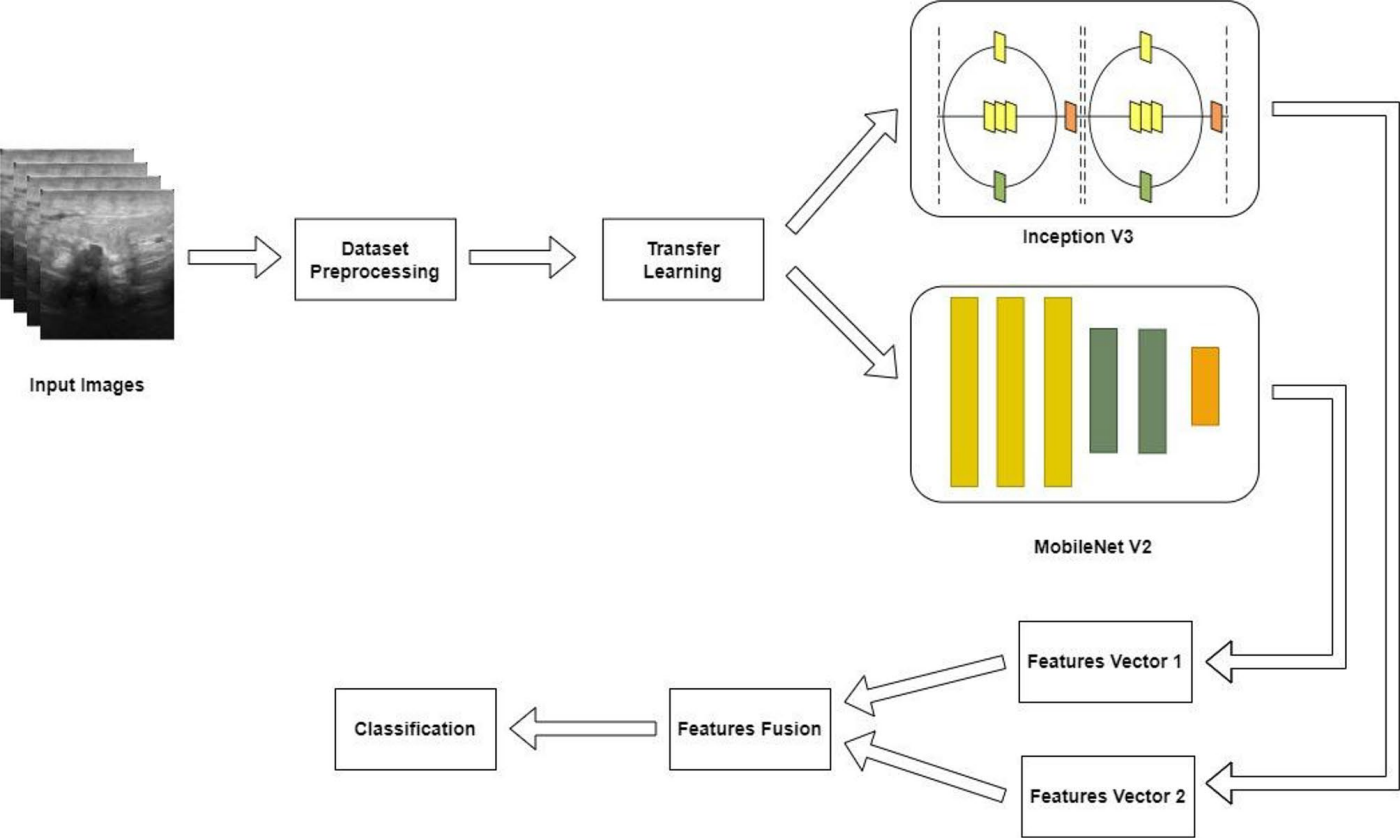
Methodology for Classification of Breast Ultrasonography

The initial step involved preprocessing the images which is followed by applying the transfer learning techniques to extract relevant features using the Inception V3 and MobileNet V2 models. Next, a serial-based fusion approach was employed to combine the extracted features which enhanced the model capability to capture the enriched patterns. The fused features are then used for classification in the final step. The overall methodology is illustrated in Fig. 2.

In the pre-processing step, the number of images is increased through the data augmentation task and the image quality is also enhanced.

#### Dataset preprocessing

Images from breast ultrasonography are included in the dataset, which is utilized for testing and training. A few preparation processes must be completed before the dataset is passed to the models. The steps taken as part of the dataset preparation are listed below:

1. Initially, the dataset is divided into various ratios for training and testing, i.e. 90/10, 80/20, 70/30, and 60/40. This step helps the model to generalize the data and provide the actual performance of the model using variation in the train-to-test ratio.
2. Images are resized to match the models’ input dimensions.
3. A special filter is applied to remove additional information from the images, such as lines etc. In this step, we used a long horizontal probing element to remove the vertical lines from the images and a long vertical probing element to remove the horizontal lines from the images.
4. Data augmentation is applied using geometric transformations. The geometric augmentations applied include rotation with multiple values, scaling, shifting width and height, and zooming in as well as out. After applying data augmentation, images increased to 500 images in each class.
5. To ensure that the pixel values fall within a range that works with the selected model, normalization is applied. While pre-trained models are designed to handle images in the range of (0, 255), scratch CNN models are built to use input in the range of (0, 1) floating point.
6. The contrast of the images is enhanced and stretched using the gray-level contrast stretching technique.
7. It was confirmed that preprocessing methods did not alter the dataset images and that they remain relevant.

#### Pre-trained deep models

Because of their excellent performance over the last several years—state-of-the-art networks have gone from having ten layers to over a thousand layers—CNNs have become increasingly complex. By using some of these cutting-edge architectures that have already been trained on ImageNet, we applied transfer learning from natural images to images from breast cancer ultrasonography in this study. The main advantage of MobileNet V2 is that it handles the gradient vanishing problem much better; moreover, it is also a lightweight model. Furthermore, the motivation for choosing the Inception V3 model is that it also reduces the parameters set and is also helpful in the case of smaller datasets.

#### Inception V3

Research suggests that the Inception V3 model may be effectively utilized for the identification and categorization of novel images by modifying the fully connected layer design and preserving the convolution layer parameters. On the ImageNet dataset, the image recognition model Inception V3 has demonstrated accuracy levels above 78.1%. The model is the result of several concepts that have been developed over time by various scholars.

In terms of object recognition, the Inception V3 model performs better than GoogleNet’s Inception V1. The enhanced Inception module, the classifier, and the fundamental convolutional block make up the Inception V3 model. For feature extraction, the fundamental convolutional block that alternates between convolutional and max-pooling layers is utilized. The improved Startup module is based on Intra-Mesh, which further cascades the convolution results of each branch and performs multi-scale convolution in parallel. The auxiliary group is used to improve gradient convergence and produce consistent training results while also minimizing overfitting and underfitting problems. Inception V3 mainly uses a 1*1 convolution kernel to reduce the number of unique channels and speed up training. Additionally, dividing the large convolution into smaller convolutions reduces the computational cost and number of parameters. In conclusion, Inception V3’s state-of-the-art object identification performance is a result of its distinctive Inception architecture. Therefore, this approach is frequently employed for transfer learning.

**Fig. 3:**
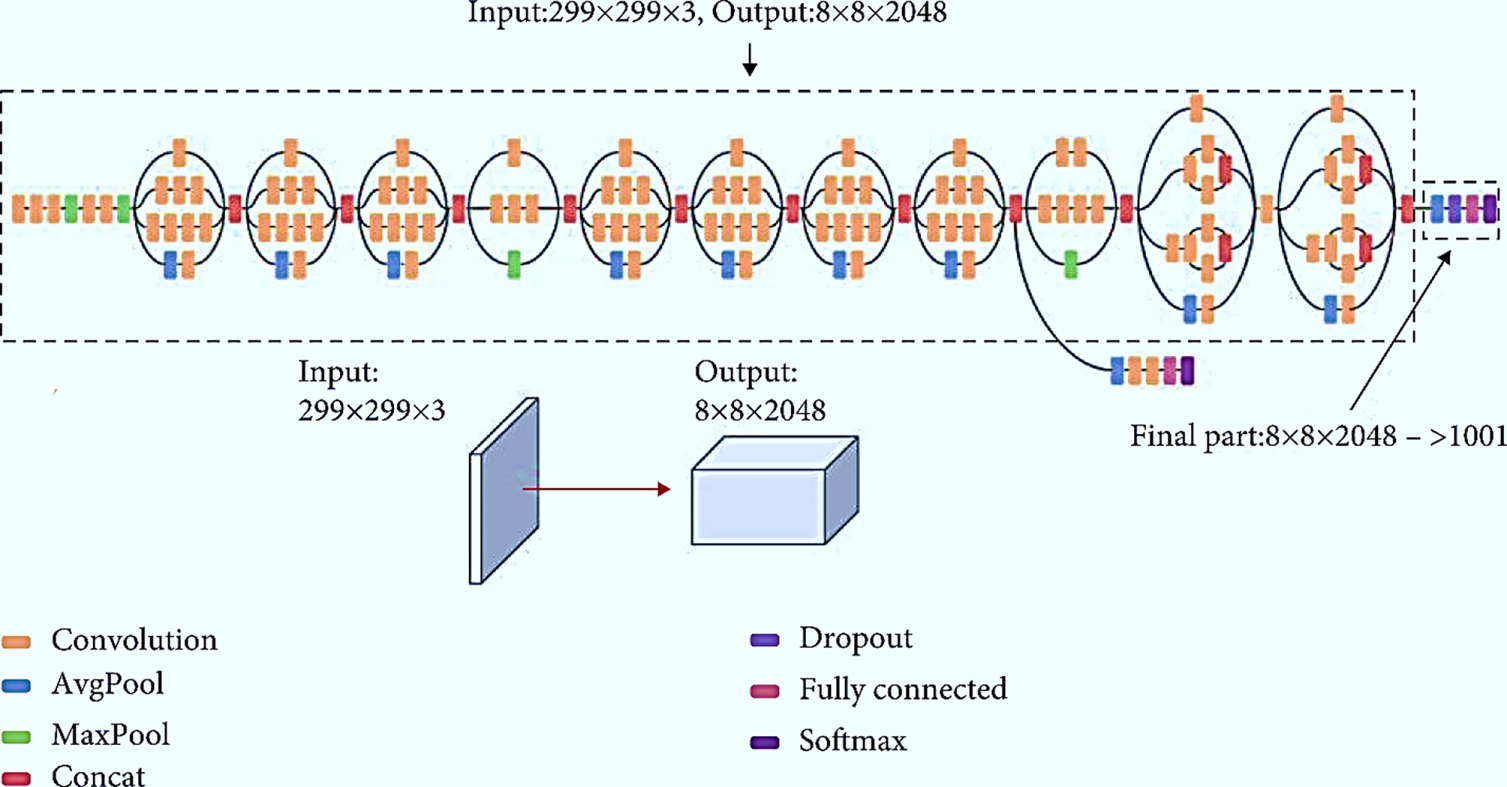
Inception V3 Architecture. The model takes an input in the format 299 × 299 × 3 and outputs the 8 × 8 × 2048. Inception V3 introduced the factorized 7 × 7 convolutions as well as batch normalization in the auxiliary classifier and performed label smoothing

Basic convolutional blocks enhanced Inception modules, and task-specific classifiers are cascaded based on the Inception-v3 model. Particularly low-performance maps were learned by the ensemble method using 1 × 1 and 3 × 3 kernels. In the initial module, the multi-scale feature representation is combined and fed to an auxiliary classifier using a set of convolution kernels (i.e. 1 × 1, 1 × 3, 3 × 1, 3 × 3, 5 × 5, 1 × 7, and 7 × 7 filters to improve convergence). Eleven inception modules act as a linked process and transform multiple performance vectors into a single vector. Finally, the Softmax classifier produces a heat vector. Figure 3 shows the Inception v3 architecture. The model extracts a total of 2048 features.

#### MobileNet V2

MobileNet is a CNN-based model that is widely used for image classification, in contrast to MobileNet V2. The primary benefit of utilizing the MobileNet architecture is that, in comparison to the traditional CNN model, it requires significantly less computing power, which makes it appropriate for use with mobile devices and PCs with less processing power. The convolution layer of the MobileNet model, which is a streamlined structure, is useful for differentiating details based on two controllable parameters that efficiently flip between latency and accuracy. Reducing the size of the network is a benefit of the MobileNet architecture.

The architecture of MobileNet is as effective while using as few resources as possible, including Palmprint Recognition. MobileNet is designed in a depth-wise manner. The basic structure is built on several abstraction layers, which are a part of various convolutions that appear to be the quantized configuration that thoroughly evaluates the difficulty of a typical problem. Point-wise complexity is the complexity of a 1 × 1 convolution. In-depth platforms are made with abstraction layers that pass through a conventional rectified linear unit (ReLU) and feature deep structural elements. To reduce the dimensionality of the input image and the internal representation of each layer, the resolution multiplier variable 𝜔 is applied.

Depending on the context, two hyperparameters, width multipliers and a resolution multiplier help translate the ideal viewing area into a more accurate estimate. The recommended input image size in the model is 224 × 224 × 3. This value must be greater than 32. It can be seen that there are three dimensions as the third value. The size of each of the 32 filters in the design is 3 × 3 × 3 × 32.

**Fig. 4:**
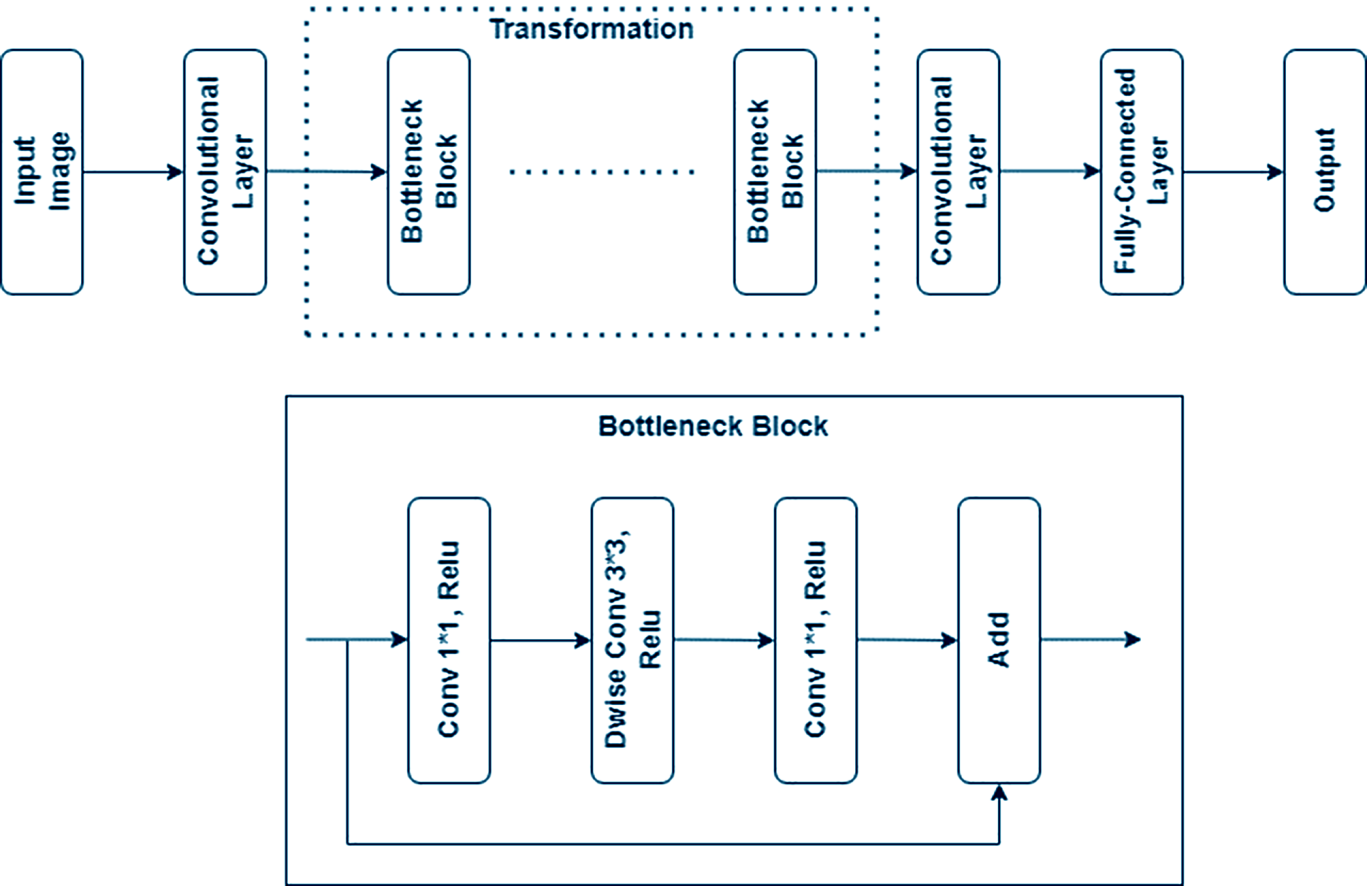
MobileNet V2 Architecture. The model takes an input of 224 × 224 × 3, and it is based on inverted residual connections between the bottleneck layers. The model in intermediately uses the depth-wise convolutions as a source of non-linearity

The idea behind MobileNet architectures is to replace complex convolutional layers with simpler ones. Each layer consists of a size 3 × 3 convolutional layer that buffers the input data and a size 1 × 1 pointwise convolutional layer that uses these filtered parameters to create a new component, as seen in Fig. 4. Simplifying the model and making it quicker than the standard convolutional model is the idea outlined above. There are 1,210 features that have been extracted from this model in total.

After the features of both models are extracted, these features are fused using a serial-based approach. The primary benefit of serial-based fusion is that it produces significant information reduction and enables real-time processing. The total features after fusion are 2,458. For reliable prediction, these fused features are then fed through a few classifiers, including Softmax, linear support vector machine (SVM), and Bayesian. The Softmax classifier utilizes cross-entropy loss for its computations. The Softmax classifier is named after the Softmax function, which transforms raw class scores into normalized positive values that sum to one, making it possible to apply cross-entropy loss effectively. SVMs are effective across a wide range of tasks because they can handle high-dimensional data and capture non-linear relationships efficiently. The success of SVMs lies in their ability to find the optimal hyperplane that maximizes the separation between different classes in the dataset. Bayesian classifiers rely on Bayes’ decision theory and use fundamental statistical principles. The core idea is that if the class is known, the values of the other characteristics may be predicted. Conversely, if the class is unknown, Bayes’ rule can be applied to predict the class label based on the provided feature values. These classifiers employ probabilistic models to determine the class label for new samples.

## Results and discussion

The dataset is split into various train-to-test ratios to facilitate experimentation. Keras, PyTorch, and Matplotlib are the primary tools and libraries used in this process. This study evaluates the proposed method using several performance criteria commonly used in breast ultrasonography categorization techniques. Accuracy and F-measure are the performance measures. Accuracy is a reliable assessment metric for evenly distributed, non-skewed classification tasks without any class imbalance. Generally speaking, accuracy can dangerously show exaggerated and overly optimistic results, particularly in imbalanced datasets. Since the F-measure is the harmonic mean of recall and accuracy, it maintains the classifier’s balance between the two [45].

The results are collected on different train-to-test ratios. Initially, results are collected on a 90 to 10 train/test ratio as shown in Table 1. The best results are achieved using the Softmax classifier with 81.26% F-measure and 82.31% accuracy. However, the Bayesian classifier produced the second-best results.

**Table 1:** Classification performance on 90/10 Train/Test ratio

	F-Measure	Accuracy
Softmax	**81.26%**	**82.13%**
Linear SVM	71.82%	70.32%
Bayesian	75.33%	78.61%

The results collected on an 80 to 20 train/test ratio are shown in Table 2. The best results are achieved using the softmax classifier with 83.71% F-measure and 85.62% accuracy. The performance of the Bayesian classifier can’t be overlooked as it produced the second-best results.

**Table 2:** Classification performance on 80/20 Train/Test ratio

	F-Measure	Accuracy
Softmax	**83.71%**	**85.62%**
Linear SVM	72.02%	73.19%
Bayesian	77.46%	79.41%

The results obtained on a 70 to 30 train/test ratio are shown in Table 3. The best results are achieved using the Softmax classifier with 93.99% F-measure and 95.82% accuracy. In a 70/30 ratio distribution, we can see that linear SVM produced the second-best results, and the results are notable; therefore, the performance of this classifier cannot be ignored.

**Table 3:** Classification performance on 70/30 Train/Test ratio

	F-Measure	Accuracy
Softmax	**93.99%**	**95.82%**
Linear SVM	89.63%	91.29%
Bayesian	87.77%	89.01%

The results obtained on a 60 to 40 train/test ratio are shown in Table 4. The best results are achieved using the Softmax classifier with 83.99% F-measure and 95.82% accuracy. In a 70/30 ratio distribution, we can see that linear SVM produced the second-best results.

**Table 4:** Classification performance on 60/40 Train/Test ratio

	F-Measure	Accuracy
Softmax	**84.67%**	**86.64%**
Linear SVM	78.74%	80.02%
Bayesian	76.36%	77.33%

As we have collected the results on different train/test ratios, we can see that the results achieved with a 70/30 train/test ratio are the best, while those with a 90/10 train/test ratio are the worst. This is because the model might be overfitted with a 90/10 train/test ratio. The best results are achieved with a 70/30 train/test ratio because the model gets the best fit and provides a perfect balance between bias and variance. This happens because the 70/30 split shows its effectiveness by training the model on a large portion of the dataset while still maintaining sufficient data for validation/testing. Moreover, we can see that the softmax classifier performed best overall. The softmax classifier provides a well-defined probability distribution as it reacts to low simulations with a normal distribution and high simulations with near to zero or one.

**Fig. 5:**
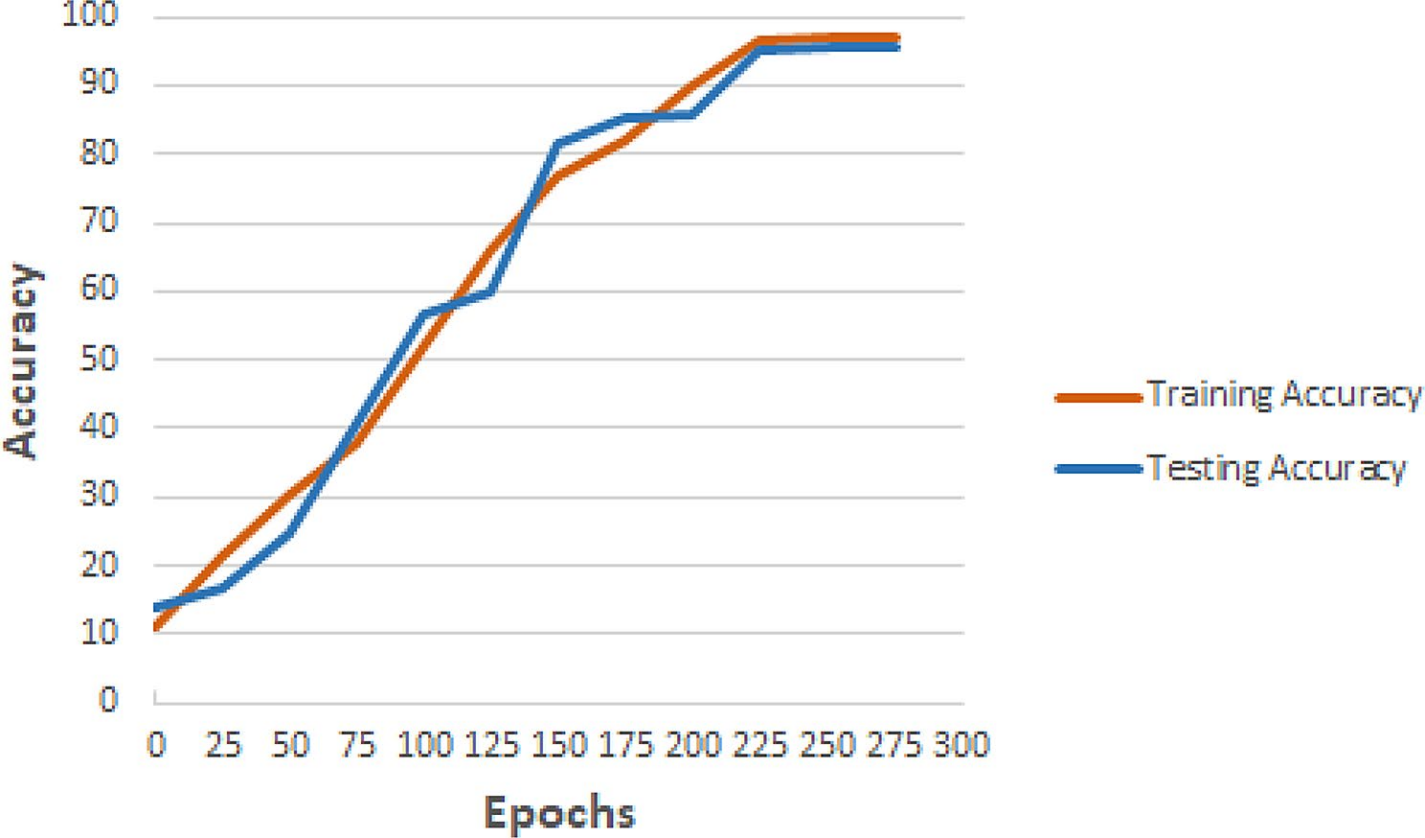
Accuracy Comparison on 70/30 Train/Test Ratio

To compare the train and test accuracies among the 70/30 train/test ratio, which also provided the best results, a comparative graph is shown in Fig. 5. Upon analyzing the graph, it is evident that initially, the model accuracy increased along with epochs. Moreover, training and testing accuracies did not differ significantly throughout the training process, which shows the model is generalizing well. Another thing to note is that the model accuracies remained constant after 250 epochs so it is justified to stop the training at 300 epochs otherwise model may start overfitting on the data.

### Ablation study

To compare the results on individual models, we have performed ablation studies. Initially, we gathered the results separately for both Inception V3 and MobileNet V2 models. Table 5 shows the classification results obtained on the Inception V3 model. It can be seen that accuracy is not up to par.

**Table 5:** Classification performance on Inception V3 Model

	F-Measure	Accuracy
Softmax	**80.12%**	**80.73%**
Linear SVM	70.01%	70.99%
Bayesian	72.88%	74.32%

The classification performance on the MobileNet V2 model is shown in Table 6.

**Table 6:** Classification performance on MobileNet V2 Model

	F-Measure	Accuracy
Softmax	**78.67%**	**79.14%**
Linear SVM	72.42%	73.67%
Bayesian	71.22%	72.19%

The results without the preprocessing steps are shown in Table 7 which provides a detailed analysis and highlights the need for the steps.

**Table 7:** Classification performance without Preprocessing Step

	F-Measure	Accuracy
Softmax	**72.15%**	**72.39%**
Linear SVM	69.83%	70.02%
Bayesian	68.54%	70.41%

**Fig. 6:**
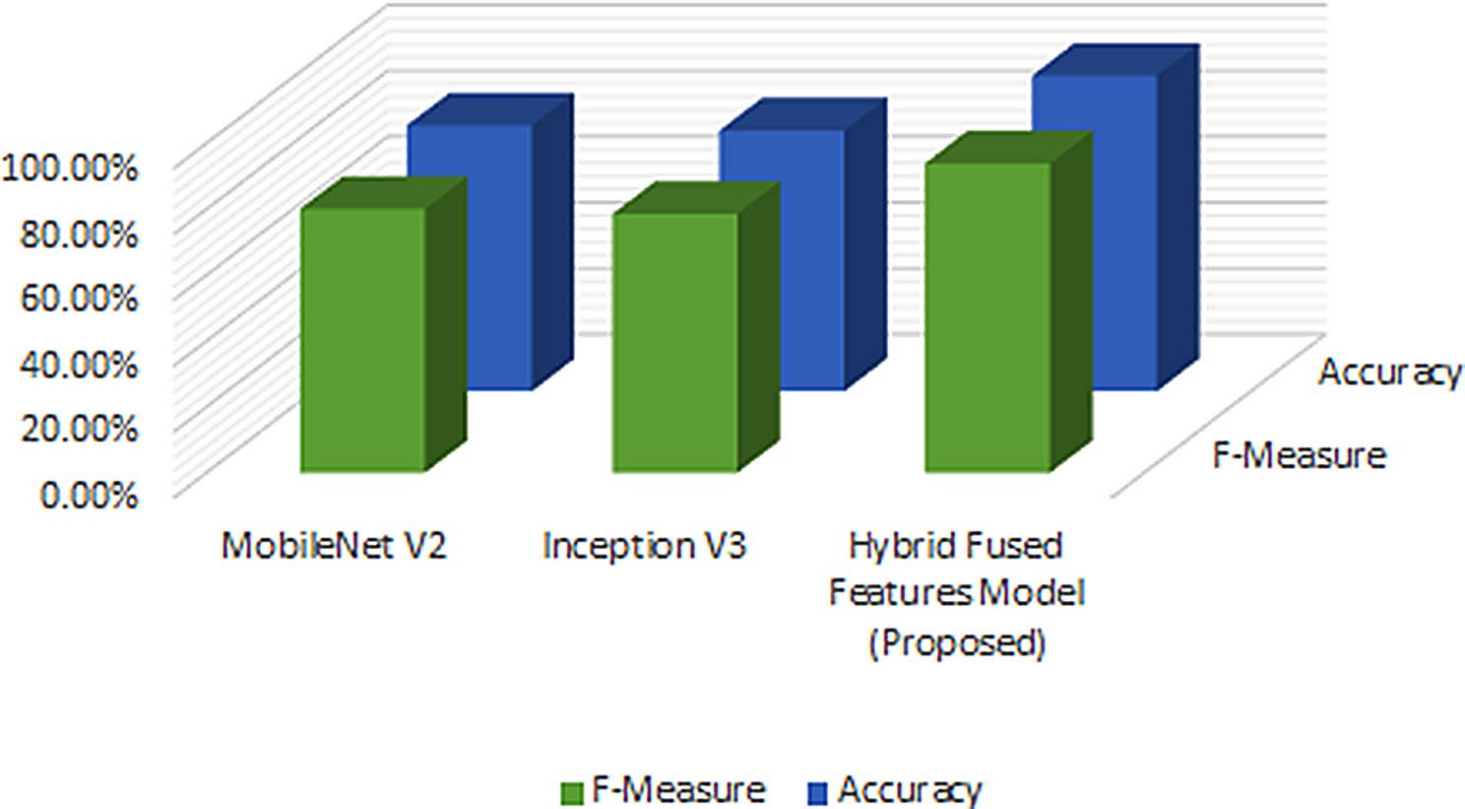
Classification Performance Comparison

Fig. 6 illustrates the accuracy comparison obtained in this work. It is evident that after feature fusion, the performance of the model improved significantly. The model can be used to explore further research and for initial clinical trials. In future work, exploring further research as well as improving the explainability of the model may be considered.

## Conclusion

Breast cancer has a high occurrence worldwide and is life-threatening. Computer-aided diagnosis greatly helps radiologists and reduces their workload. Therefore, in this research, we have proposed a hybrid CNN system for the diagnosis of benign and malignant breast cancer lesions. The models InceptionV3 and MobilenetV2 serve as the foundation for the hybrid framework. These models’ characteristics are extracted and concatenated individually. As a result, more features are utilized. Finally, various classifiers are applied to the classification task. The model has achieved the best results using a softmax classifier and a 70/30 train/test ratio. In future work, we will explore explainable AI techniques on the data present the findings. We will also investigate multi-modal data.

## Data Availability

The dataset analyzed during the current study is available in the [https://www.kaggle.com/datasets/aryashah2k/breast-ultrasound-images-dataset] repository.
